# Comparison of the onset time between 0.375% ropivacaine and 0.25% levobupivacaine for ultrasound-guided infraclavicular brachial plexus block: a randomized-controlled trial

**DOI:** 10.1038/s41598-021-84172-2

**Published:** 2021-02-25

**Authors:** Ha-Jung Kim, Sooho Lee, Ki Jinn Chin, Jin-Sun Kim, Hyungtae Kim, Young-Jin Ro, Won Uk Koh

**Affiliations:** 1 Department of Anesthesiology and Pain Medicine, Asan Medical Center, University of Ulsan, 88, Olympic-ro 43-gil, Songpa-gu, Seoul, 05505 Korea; 2 Department of Anesthesia and Pain Medicine, College of Medicine, International St. Mary’s Hospital, Catholic Kwandong University, Incheon, 22711 Korea; 3 Department of Anesthesia, Toronto Western Hospital, University of Toronto, Toronto, ON Canada

**Keywords:** Outcomes research, Randomized controlled trials

## Abstract

At centers with pressure on rapid operating room turnover, onset time is one of the important considerations for choosing a local anesthetic drug. To hasten the onset of the block, higher concentrations of local anesthetics are sometimes used. However, the use of diluted local anesthetics may be safer. Therefore, we aimed to compare the onset times of equipotential levobupivacaine and ropivacaine at low concentrations for infraclavicular brachial plexus block. Adult patients undergoing upper extremity surgery under ultrasound-guided infraclavicular brachial plexus block at our center were randomly allocated to the levobupivacaine and ropivacaine groups. Infraclavicular brachial plexus block was induced with 0.25% levobupivacaine or 0.375% ropivacaine depending on the assigned group. The degrees of sensory and motor blockade were assessed for 40 min after the administration of local anesthetics. A total of 46 patients were included in the analysis. Infraclavicular brachial plexus block with 0.25% levobupivacaine and 0.375% ropivacaine provided sufficient surgical anesthesia. The sensory onset time of 0.375% ropivacaine was shorter than that of 0.25% levobupivacaine (group R, 15 [15.0–22.5] min; group L, 30 [17.5–35.0] min, *p* = 0.001). There were no significant differences in other block characteristics and clinical outcomes between the two groups. Thus, when a quicker block onset is required, 0.375% ropivacaine is a better choice than 0.25% levobupivacaine.

**Trial registration** ClinicalTrials.gov (NCT03679897).

## Introduction

Infraclavicular brachial plexus block provides sufficient anesthetic and analgesic effect for lower arm surgery^1^. Infraclavicular approach is not only advantageous for inserting a perineural catheter, but also has a short procedure time compared to other approaches including supraclavicular and axillary approaches^2,3^. Therefore, ultrasound-guided infraclavicular brachial plexus block has been increasingly used since the first report in 2000^4^.

Although peripheral nerve blocks have several benefits over general anesthesia, some clinicians are still reluctant in using it because of the relatively long latency period. At centers with pressure on rapid operating room turnover, onset time is one of the important considerations for choosing a local anesthetic drug. The most commonly used local anesthetic agents in peripheral nerve block are levobupivacaine and ropivacaine, both of which have long duration of action and are preferred for prolonged surgery and postoperative pain control^5^. However, these two drugs have a considerable disadvantage of relatively delayed onset^5^.

To hasten the onset of local anesthetic agent, some anesthesiologists increases the concentration of local anesthetics^6^. However, higher concentration of local anesthetics not only have been associated with increased direct neurotoxicity to the neurons but the volume must be carefully selected to avoid exceeding the maximal dose^7^. Finding a lower concentration local anesthetic agent with quicker onset is important to anesthesiologists, especially in centers without a preparation room for performing blocks. However, previous literature usually reported comparing the sensory onset time of levobupivacaine and ropivacaine at relatively higher concentrations^8,9^. In addition, most studies compared these two drugs at the same concentration, although it has turned out that the potency of the drugs is not the same^10^. Therefore, in this present study, we aimed to compare the onset times of sensory block with equipotential 0.25% levobupivacaine and 0.375% ropivacaine. In addition, other block characteristics and clinical outcomes were also analyzed.

## Results

### Study population

**Figure 1:**
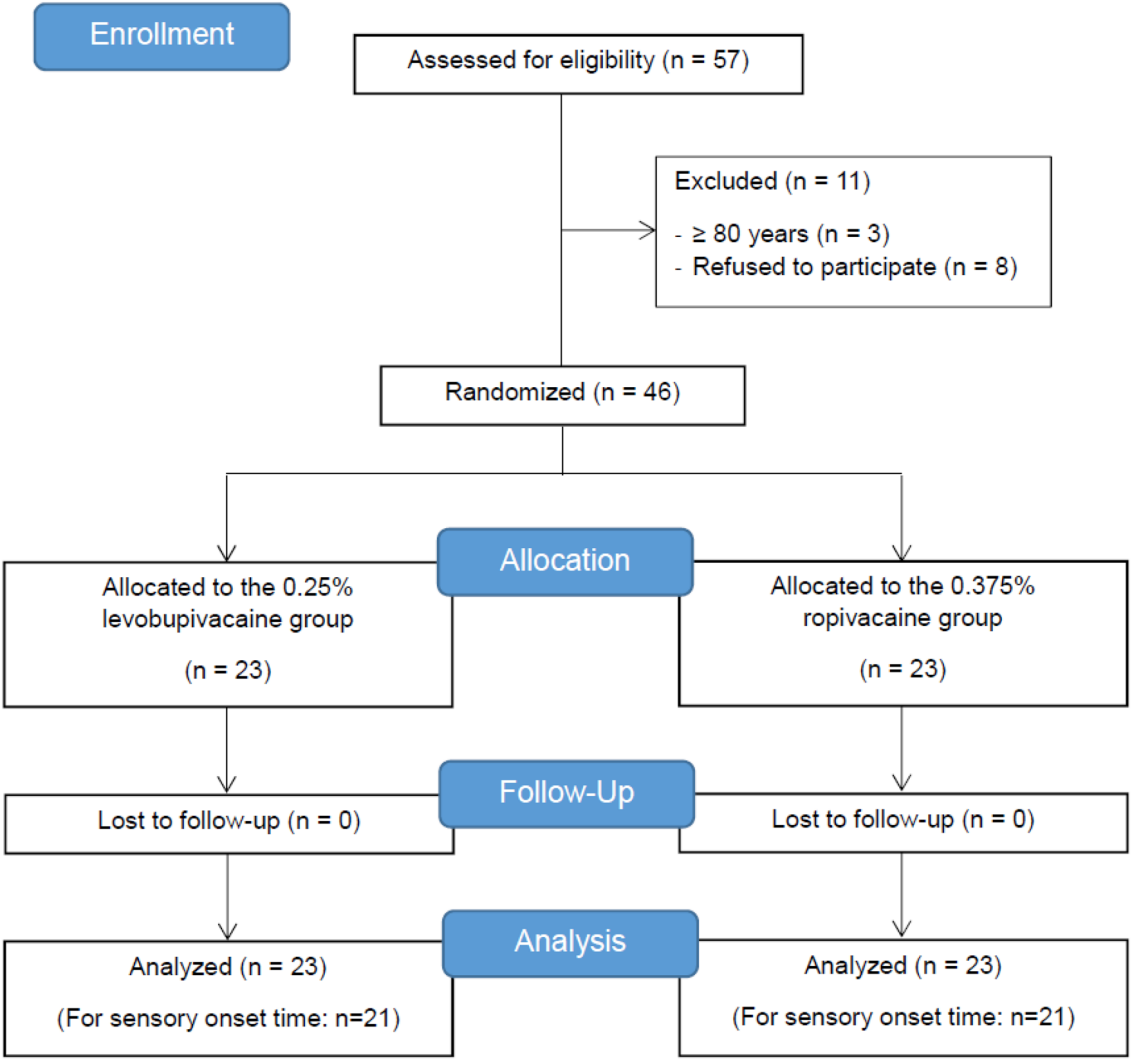
Flow diagram presenting enrollment, intervention allocation, follow-up and data analysis.

Between September 2018 and December 2018, 57 patients who were scheduled to undergo upper extremity surgery, with brachial plexus block as the main anesthetic method, were screened for eligibility. Among them, 3 patients were older than 80 years and 8 patients disagreed to participate in this study, and they were excluded. This study was conducted on 46 patients and there was no drop-out. Therefore, a total of 46 patients were included in the final analysis (Fig. 1). Table 1 shows the baseline characteristics of the study patients and surgery. There was no difference between the two groups.

**Table 1:** Demographic data of the patients.

	Group L (n = 23)	Group R (n = 23)	*p*
Age (y)	53.5 ± 16.2	56.6 ± 14.9	0.499
Sex (F:M)	7:16 (30.4%:69.6%)	12:11 (52.2%:47.8%)	0.231
Height (cm)	166.4 ± 9.7	162.6 ± 8.6	0.157
Weight (kg)	68.9 ± 12.2	65.1 ± 11.5	0.290
BMI (kg/m^2^)	24.7 ± 2.7	24.6 ± 3.9	0.913
**Surgery**			
Bone:soft tissue	17:6 (73.9%:26.1%)	14:9 (60.9%:39.1%)	0.529
Elbow:forearm:hand	3:10:10 (13.0%:43.5%:43.5%)	4:13:6 (17.4%:56.5%:26.1%)	0.530
Duration of surgery (min)	76.7 ± 33.0	69.1 ± 32.1	0.438

Data are presented as mean ± standard deviation, or number (%).

### Onset time of sensory and motor block

**Figure 2:**
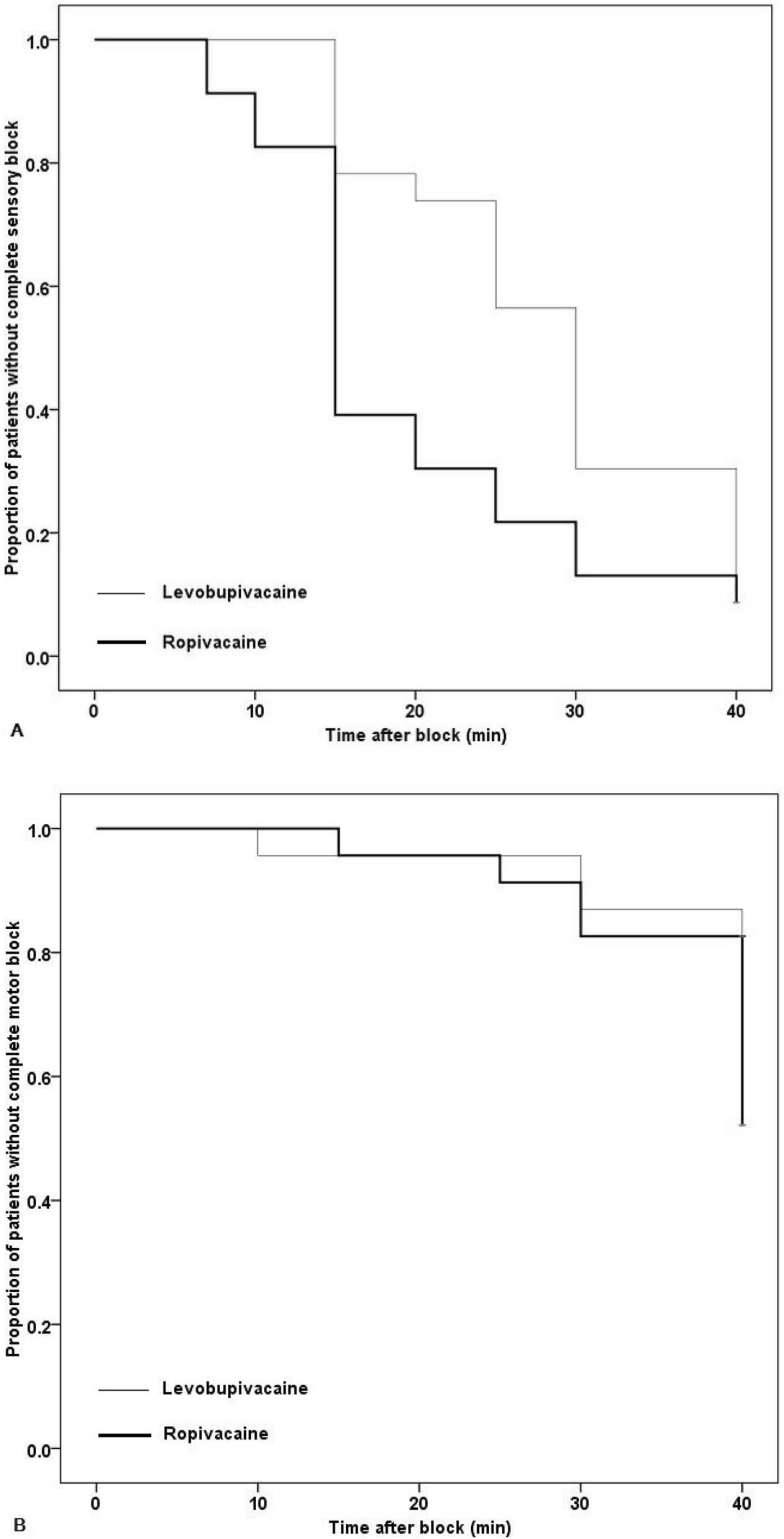
Kaplan–Meier curve of proportion of patients who achieved (**A**) a complete sensory block and (**B**) a complete motor block after infraclavicular brachial plexus block. (**A**) log-rank test, *p* = 0.032; (**B**) log-rank test, *p* = 0.045.

The median onset time of sensory block was significantly shorter in group R than in group L (group R, 15 [15.0–22.5] min; group L, 30 [17.5–35.0] min, *p* = 0.001). Table 2 shows the success rate of complete sensory and motor blockade at each time point in the two groups. The success rates at 15, 20, and 25 min elapsed from the drug injection were significantly higher in group R (*p* = 0.016, 0.007, and 0.033, respectively). However, there was no difference in success rate after 30 min, and both groups had a success rate higher than 90% at 40 min. The Kaplan–Meier survival curve for the proportion of patients who achieved a complete sensory block revealed that the complete sensory block incidence was higher in group R than in group L (log-rank test, *p* = 0.032, Fig. 2A).

**Table 2:** The success rate of complete sensory and motor blockade at each time point.

Time (min)	Complete sensory block			Complete motor block		
	Group L (n = 23)	Group R (n = 23)	*p*	Group L (n = 23)	Group R (n = 23)	*p*
2	0 (0)	0 (0)	> 0.999	0 (0)	0 (0)	> 0.999
5	0 (0)	0 (0)	> 0.999	0 (0)	0 (0)	> 0.999
7	0 (0)	2 (8.7)	0.489	0 (0)	0 (0)	> 0.999
10	0 (0)	4 (17.4)	0.109	1 (4.3)	0 (0)	> 0.999
15	5 (21.7)	14 (60.9)	0.016	1 (4.3)	1 (4.3)	> 0.999
20	6 (26.1)	16 (73.9)	0.007	1 (4.3)	1 (4.3)	> 0.999
25	10 (43.5)	18 (78.3)	0.033	1 (4.3)	2 (8.7)	> 0.999
30	16 (69.6)	20 (87.0)	0.284	3 (13.0)	4 (17.4)	> 0.999
40	21 (91.3)	21 (91.3)	> 0.999	4 (17.4)	11 (47.8)	0.0574

Data are presented as number (%).

The success rates of motor blockade at each time point demonstrated no difference in the two groups. However, the Kaplan–Meier curve, showing the proportion of patients who achieved a complete motor block, demonstrated that the complete motor block proportion was higher in group R than in group L (log-rank test, *p* = 0.045, Fig. 2B). Because the success rate of the motor block at 40 min was too low in both groups, the median onset time of the motor block was not analyzed.

### Other block-related characteristics and clinical outcomes

The data associated with block performance and results are shown in Table 3. No difference was found in the performance time and total administered volume of local anesthetics. In addition, there were no differences between the rates of conversion into general anesthesia and the requirements for supplemental anesthetics or analgesics. The total administered doses of intraoperative intravenous dexmedetomidine of the two groups were also not different. No local anesthetic systemic toxicity occurred in both groups. The durations of the sensory and motor blockade of the two groups and the indicators of patient satisfaction were also not different (Table4).

**Table 3:** Block performance details and outcomes of the block.

	Group L (n = 23)	Group R (n = 23)	*p*
Performance time (min)	2.0 [2.0–4.0]	2.0 [2.0–3.0]	0.204
Total administered volume of local anesthetics (mL/kg)	0.51 [0.48–0.54]	0.50 [0.48–0.56]	0.749
Total administered volume of local anesthetics (mL)	34.0 [32.0–38.0]	32.0 [30.0–36.0]	0.131
Conversion into general anesthesia	0 (0.0%)	1 (4.3%)	> 0.999
Requirements of supplemental anesthetics or analgesics	4 (17.4%)	3 (13.0%)	> 0.999
Sevoflurane	3 (13.0%)	0 (0.0%)	0.233
Fentanyl	4 (17.4%)	3 (13.0%)	> 0.999
Total administered dose of dexmedetomidine (mcg)	127.8 ± 40.7	111.9 ± 34.0	0.156
Local anesthetics systemic toxicity	0 (0.0%)	0 (0.0%)	> 0.999

Data are presented as mean ± standard deviation, median [interquartile range], or number (%).

**Table 4:** Block duration and patient satisfaction.

	Group L (n = 23)	Group R (n = 23)	*p*
Duration of sensory block (min)	996.0 [818.0–1230.5]	899.0 [816.5–1038.5]	0.267
Duration of motor block (min)	1044.0 [937.5–1201.5]	1046.0 [851.5–1112.0]	0.277
Patient satisfaction	5.0 ± 2.0	5.3 ± 1.7	0.573

Data are presented as mean ± standard deviation or median [interquartile range].

## Discussions

In this study, infraclavicular brachial plexus block with 0.25% levobupivacaine and 0.375% ropivacaine provided sufficient surgical anesthesia, and we found that 0.375% ropivacaine had a shorter onset time of sensory and motor block than 0.25% levobupivacaine. However, there were no significant differences in other block characteristics and clinical outcomes between the two groups.

Levobupivacaine and ropivacaine belong to the amino-amide local anesthetics and are optically pure S-enantiomers of bupivacaine. They have very similar pharmacologic characteristics; in particular, they have the same ionized constant (pKa = 8.1), which is known to be associated with onset time^11^. However, the liposolubility of levobupivacaine and ropivacaine are 30 and 2.8, respectively^5^. The disparity between the two drugs is a consequence of the fact that ropivacaine contains pipecoloxylidine with a 3-carbon side-chain, whereas levobupivacaine has a 4-carbon side-chain^12^. Previous in vitro studies showed that higher lipid solubility hastens the onset time in isolated nerve fibers, although these results are not necessarily consistent in the clinical setting and many authors have asserted that it should be appreciated comprehensively with other factors^11^. The difference in the liposolubility between levobupivacaine and ropivacaine might somewhat explain our results.

Some previous studies comparing the onset time of levobupivacaine and ropivacaine did not find any difference^13,14^. However, these studies compared the onset of levobupivacaine and ropivacaine at the same concentrations^13,14^. Brau et al. identified that levobupivacaine was nearly 50% more potent than ropivacaine for blocking the neuronal tetrodotoxin-resistant sodium channels^15^. Moreover, a recent systematic review article analyzing clinical studies demonstrated that levobupivacaine was more potent than ropivacaine for peripheral nerve blocks, and the ratio of the potency of the ropivacaine to that of levobupivacaine was approximately 2:3^5,10^. Thus, this study is meaningful, as we compared the onset times of levobupivacaine and ropivacaine at different concentrations of equal potency, taking into account that the potencies of the two drugs at the same concentrations are different.

The widely accepted local anesthetic concentration range for surgical anesthesia is 0.5–0.75% for both levobupivacaine and ropivacaine. In terms of local anesthetic volume, Chin et al. reported that 20–40 mL of local anesthetics are generally required for US-guided infraclavicular brachial plexus block^1^. However, the maximal recommended doses of levobupivacaine and ropivacaine are 2 mg/kg and 3 mg/kg, respectively^16^, and considering the maximal dose, the use of highly concentrated local anesthetics of sufficient volume increases the likelihood of systemic toxicity. To minimize the risk of systemic toxicity while achieving a successful block with a sufficient volume of local anesthetics, it is advantageous to use drugs at lower concentrations. Mosaffa et al. asserted that the higher volume of the diluted local anesthetic agent accelerated the sensory and motor onset without an effect on the success rate for infraclavicular brachial plexus block^17^. A review article indicated that the concentration of local anesthetics was unlikely relevant to block onset, success, and duration, but the mass of local anesthetics was the most determinant factor in the peripheral nerve block^18^. In this study, our regimen, using levobupivacaine and ropivacaine of lower concentrations, showed sensory block success rates higher than 90% at 40 min elapsed from the drug injection without any complication in both groups.

To our best knowledge, this present study was the first clinical trial that compared the onset time of 0.25% levobupivacaine and 0.375% ropivacaine for brachial plexus block as surgical anesthesia. The median value of 0.375% ropivacaine sensory onset time in this study was 15 min, which was consistent with previous studies that evaluated the sensory onset time of 0.375% ropivacaine in brachial plexus block^19^. In a previous study by Wank et al. 0.375%, ropivacaine provided a complete motor block 50 min after the block only in 30–50%^20^. Our study also showed that less than 50% of cases reached the complete motor block 40 min after the injection of 0.375% ropivacaine. On the contrary to the ropivacaine, a few studies have been conducted on surgical anesthesia with 0.25% levobupivacaine. One study reported that axillary brachial plexus block with 0.25% levobupivacaine required approximately 25 min for complete sensory block, and only 27% had a complete motor block^21^. These results were quite similar to our results. Based on the results of this study, although 0.25% levobupivacaine could provide sufficient surgical anesthesia, it may not be the most appropriate drug considering the onset time, especially in clinical environments where rapid turnover is required. In addition, there was no notable benefit over 0.375% ropivacaine considering other block-related characteristics. Therefore, 0.375% ropivacaine may be a better choice than 0.25% levobupivacaine in clinical settings.

Rapid onset of action is important in the clinical setting for several reasons when a surgical peripheral nerve block is applied. The rapid action of local anesthetics will improve the efficiency in operating room management, reduces surgeon’s concerns for the time consumption, and increases the patient satisfaction by reducing anxiety. The results of current study suggest to use 0.375% ropivacaine for infraclavicular brachial plexus block rather than 0.25% levobupivacaine. However, in case of requirement of rapid onset of both complete sensory and motor block is required, co-administration of other local anesthetics with a quicker onset or the addition of adjuvants could be helpful^22,23^. Considering that systemic toxicity of local anesthetics are additive, the total volume of mixed local anesthetics should be carefully decided regarding individual patient characteristics^24^.

There are several research results about infraclavicular brachial plexus block that have been recently published^25–31^. For the infraclavicular block, there were various regimens of local anesthetics in terms of type, concentration, adjuvant and volume of local anesthetics. There were also various approaches including parasagittal, costoclavicular, and retroclavicular approaches (Supplement 1). Although the block success rate reached over 90% in most of the studies, the results of the studies on onset time of the block were inconsistent and variable. For example, Vazin et al. reported that the onset time of 0.75% ropivacaine using parasagittal approach was 30 min, while Abhinava et al. demonstrated that the onset time of 0.5% ropivacaine using parasagittal approach was only 6.43 min^25,31^. In addition, there was little research about the characteristics of infraclavicular block using levobupivacaine. Further systematic and comprehensive analysis about infraclavicular block using various drug regimens and approaches is required in the near future.

This study had limitations. First, we evaluated the extent of block achieved for only 40 min. Because this study was conducted in a high-volume tertiary center, where turnovers occur very quickly, we could not observe the entire period until complete sensory and motor block was achieved. However, since most centers are under pressure to improve efficiency in the management of the operating room, time consumption longer than 40 min for anesthesia may be difficult to accept. Besides, the complete motor block is not a prerequisite for surgery, and in most cases, the sensory block was complete within 40 min after the procedure. Second, the onset time we measured may not be very accurate, as the procedure took a few minutes; dual-injection rather than single-injection techniques were used and the assessment of the blockade extent was not continuously performed. However, the aim of this study was not to find out the exact onset time of each drug, but to identify the drug which shows faster sensory onset at an equipotent concentration. Moreover, we used infraclavicular block which could be performed quickly to reduce the confounding effect by the onset of the drug during the block procedure. Also, since the dual-injection technique was applied equally in the two groups, it did not affect the main result.

In conclusion, 0.375% ropivacaine showed a shorter onset time of sensory block than 0.25% levobupivacaine in infraclavicular block for upper limb surgeries. Other characteristics of the two drugs related to the block were not different. Thus, when a quicker block onset is required, 0.375% ropivacaine is a better choice than 0.25% levobupivacaine.

## Methods and materials

This prospective, double-blinded (participants and assessors), randomized controlled study was conducted at a tertiary center in Seoul, Korea. It was approved by the ethics committee of Asan Medical Center (Institutional Review Board no. 2018-1117, September, 2018), and written informed consent was obtained from all patients. This study was conducted in compliance with the 1964 Helsinki declaration and its later amendments. We registered this study on ClinicalTrials.gov (NCT03679897, registration date: 21/09/2018). The manuscript is written in accordance with Consolidated Standards of Reporting Trials (CONSORT) guidelines.

### Patients and preparation

We included patients who met the following criteria: (1) ASA physical status 1–3, (2) age between 19 and 80 years, and (3) scheduled to undergo upper extremity surgery with infraclavicular brachial plexus block at our center. Patients with neurological deficits of the upper extremity, severe coagulopathy, chronic renal failure, cardiopulmonary compromise, cerebral vascular disease, hypersensitivity to local anesthetics, or local infection at the site of the infraclavicular block were excluded. Patients who were pregnant and those who refused to participate in the clinical trial were also excluded. All the included patients were randomly allocated to two groups in a 1:1 ratio: 0.25% levobupivacaine group (Group L) and 0.375% ropivacaine group (Group R). A computer-generated randomization program (Randomization.com) was used for the random allocation, and the patients were allocated just before receiving the block. Based on the randomization, the study drug was prepared in two 20 mL-syringes by the corresponding author (WUK) of this study. For group L, 20 mL of 0.5% levobupivacaine (Chirocaine; Abbvie, Norway) was mixed with 20 mL normal saline resulting in a total of 40 mL of 0.25% levobupivacaine. For group R, 20 mL of 0.75% ropivacaine (Naropin; AstraZeneca, Australia) and 20 mL normal saline were mixed, resulting in 40 mL of 0.375% ropivacaine. The syringes containing the study drugs were handed to a nurse who was blinded to the group allocations in this study.

### Anesthesia

All the blocks were performed in the pre-induction room by the first author (HJK) using parasagittal approach and dual-injection technique to increase the success rate of the block^32^. After the patients arrived in the pre-induction room without any premedication, they were positioned supine. Standard monitoring, including electrocardiography, non-invasive blood pressure, and pulse oximetry were applied. Five to six liters of oxygen per minute was supplied via a simple facial mask.

Patients were positioned supine with their head turned to the contralateral side and the ipsilateral arm was kept neutral. The skin near the clavicle was disinfected and sterile drapes were applied. The high frequency 5–13 Hz linear transducer of the ultrasound machine (Logiq P9; GE health care, Nolensville, Tennessee, US) was used, and the probe was located just medial to the coracoid process in the sagittal direction. The lateral, medial, and posterior cords around the axillary artery beneath the pectoralis muscles were identified in the ultrasound view. The site of needle entry was located 1 cm medial to the coracoid process and 1 cm below the clavicle^33^. At the insertion point of the needle, 1 mL of 2% lidocaine was injected for local infiltration. A 22‐G, 60‐mm stimulating needle (Stimuplex D; B.Braun AG, Melsungen, Germany) was inserted in‐plane initially toward the posterior cord. A nerve stimulator (MultiStim SENSOR; PAJUNK GmbH Medizintechnologie, Geisingen, Germany) was used to localize the cords using an electrical current of 0.3–0.5 mA while observing the muscle twitches. After the identification of the posterior cords with the motor response, 0.2–0.3 mL/kg of the prepared study drug was administered. The needle was withdrawn to the subcutaneous tissue and redirected toward the medial cord, and an additional dose of 0.2–0.3 mL/kg was injected with the visualization of the spread of local anesthetics. Throughout the administration of the study drug, the nurse performed intermittent aspiration of the syringe to avoid inadvertent intravascular injections. In all cases, the local anesthetic agent was deposited so as to surround the medial, lateral, and posterior cord around the axillary artery in the ultrasound view. After the completion of the injection, the degrees of sensory and motor blocks were assessed for 40 min, and all the patients were moved to the operation theater. Then, the sedation with dexmedetomidine was started and maintained during the surgery. A dose of 1 mcg/kg dexmedetomidine was loaded over 10 to 15 min, followed by a continuous infusion of 0.5–1.0 mcg/kg/h. The requirement of supplementary anesthetics or analgesics and conversion to general anesthesia was determined by the attending anesthesiologist 40 min after the completion of the injection. Supplementary anesthetics or analgesics included the use of fentanyl, application of additional distal nerve block above the elbow level by anesthesiologists, or transient supplementation of sevoflurane through a facial mask. Local infiltration as a rescue block by surgeons was not allowed. When the anesthesia was insufficient for surgery despite any supplementary anesthetics or analgesics, general anesthesia was induced with sevoflurane and insertion of supraglottic airway device or endotracheal tube was performed for maintenance of general anesthesia. Supplementation of sevoflurane via inserted supraglottic airway device or endotracheal tube was considered as the conversion to general anesthesia. During the perioperative period, the anesthesiologist checked for local anesthetic systemic toxicity and other adverse effects. All the surgeries were conducted on the lower arm including the elbow, forearm, or hand with the pneumatic tourniquet. After the surgery, the patient was transferred to the post-anesthetic care unit and then taken to a general ward.

### Assessment

The degrees of sensory and motor blockade of the study patients were assessed in the distributions of the musculocutaneous, radial, median, and ulnar nerves. The assessment was performed for 40 min immediately after the completion of the drug injection (T0) by the first author who was blinded to the injected drug. During the first 10 min after the drug injection, the assessment was performed more frequently (at 2, 5, 7, 10 min elapsed from the completion of local anesthetics injection). Subsequently, evaluations were made at 5-min intervals and we defined time. A 3-point scale (2 = normal sensation, 1 = reduced, and 0 = absent) was used for grading the extent of sensory block to the pinprick stimuli in comparison to the corresponding area of the contralateral arm. Sensory onset time, which is the primary outcome of this study, was defined as the time required to achieve an absence of sensation in response to the pinprick stimuli at all distributions of the four nerves. The degree of motor block was also graded on a 3-point scale (2 = normal, 1 = reduced power, 0 = paralysis). In addition, we defined the success of complete sensory or motor block as when the sum of sensory or motor scores in each of the four nerve territories reached zero. Total performance time was defined as the time from the needle insertion to the completion of the injection. On the first postoperative day, an assessor visited the patients and questioned them regarding the duration of the sensory and motor block; the patients were surveyed for satisfaction with the block using a 7-point Likert scale^34^. All data were recorded on a standardized case report form.

### Statistics

Based on our clinical experiences, the sensory onsets of 0.25% levobupivacaine and 0.375% ropivacaine were approximately 20 ± 5 min and 15 ± 5 min, respectively. The calculated sample size, with α = 0.05 and power = 90% using a two-sample, two-sided equality test, was 21 patients in each group. Thus, we allocated 23 patients to each group considering 10% dropouts. R version 4.0.0 was used for all data analyses. Continuous data were compared using the Mann–Whitney U test or Student t-test and categorical data were analyzed with Fisher’s exact test. In the analysis of the onset times, the incomplete block cases at 40 min elapsed from the injection were excluded. In addition, the cumulative success rates of sensory and motor blocks in each group were analyzed using the Kaplan–Meier analysis, and alterations between curves were estimated by the log-rank test. A *p* value of less than 0.05 was considered statistically significant.

## Supplementary Information

Supplementary Information.
