# Supplementary material for: Comparison of the onset time between 0.375% ropivacaine and 0.25% levobupivacaine for ultrasound-guided infraclavicular brachial plexus block: a randomized-controlled trial
Source: Sci Rep. 2021 Feb 25;11:4703. doi: 10.1038/s41598-021-84172-2 (PMC7907375; doi:10.1038/s41598-021-84172-2)
Supplement: Supplementary file 1 — Supplementary Information. [file 41598_2021_84172_MOESM1_ESM.docx]

**Comparison of the onset time between 0.375% ropivacaine and 0.25% levobupivacaine for ultrasound-guided infraclavicular brachial plexus block: a randomized-controlled trial**

Ha-Jung Kim (MD, PhD)^a^, Sooho Lee (MD)^b^, Ki Jinn Chin (FRCPC)^c^, Jin-Sun Kim(MD)^a^, Hyungtae Kim(MD)^a^, Young-jin Ro(MD, PhD)^a^, and Won Uk Koh(MD, PhD)^a,*^

**Author affiliations:**

1. Department of Anesthesiology and Pain Medicine, Asan Medical Center, University of Ulsan, 88, Olympic-ro 43-gil, Songpa-gu, Seoul 05505, Korea
2. Department of Anesthesia and Pain Medicine, Catholic Kwandong University, College of Medicine, International St. Mary’s hospital, Incheon 22711, Korea
3. Department of Anesthesia, Toronto Western Hospital, University of Toronto, Toronto, Ontario, Canada

| **Title** | **Author** | **Year** | **Study design** | **LA regimen** | **Nerve localization** | **Approach** | **Sensory onset time** | **Block success** | **Duration** |
| --- | --- | --- | --- | --- | --- | --- | --- | --- | --- |
| Low-volume brachial plexus block providing surgical anesthesia for distal arm surgery comparing supraclavicular, infraclavicular, and axillary approach: a randomized observer blind trial.^25^ | Vazin et al. | 2016 | RCT | 20mL of 0.75% ropivacaine | US-guided | Parasagittal  -3 injection points: | 30 [5] minutes | 36/40 (90%) at 40 minutes | 822 [475] minutes |
| Retroclavicular vs infraclavicular block for brachial plexus anesthesia: a multi-centric randomized trial.^26^ | Blanco et al. | 2019 | RCT | 20mL of 0.5% ropivacaine + 20mL of 1.5% mepivacaine | US-guided | Retroclavicular  -1 injection point  Infraclavicular (Parasagittal)  -1 injection point | Not reported | 50/53 (94.3%)  at 30 minutes  91.1% at 30 minutes | Not reported |
| Comparison of the coracoid and retroclavicular approaches for ultrasound-guided infraclavicular brachial plexus block.^27^ | Ozturk et al. | 2017 | RCT | 25mL of 0.5% bupivacaine | US-guided | Coracoid (Parasagittal)  -1 injection point  Retroclavicular  -1 injection point | 18.2 ± 5.1 minutes  15.4 ± 6 minutes | 45/50 (90%) at 30 minutes  48/50 (96%) at 30 minutes | Not reported |
| Ultrasound-guided infraclavicular brachial plexus block: prospective randomized comparison of the lateral sagittal and costoclavicular approach.^28^ | Songthamwat et al. | 2018 | RCT | 25mL of 0.5% ropivacaine | US-guided | Lateral sagittal (Parasagittal) -2 injection points  Costoclavicular  -1 injection point | 20 [15] minutes  10 [16.25] minutes | 100% at 45 minutes  100% at 45 minutes | Not reported |
| Comparative evaluation of two approaches of infraclavicular brachial plexus block for upper-limb surgeries.^29^ | Sinha et al. | 2019 | RCT | 20mL of 0.5% levobupivacaine | US-guided | Parasagittal  -1 injection point  Retroclavicular  -1 injection point | Not reported | 57/60 (95%) at 30 minutes  57/60 (95%) at 30 minutes | Not reported |
| Comparison of ultrasound-guided supraclavicular, infraclavicular and below-C6 interscalene brachial plexus block for upper limb surgery: a randomised, observer-blinded study.^30^ | Bharti et al. | 2017 | RCT | 0.5mL/kg of 0.75% ropivacaine + 2% lignocaine-adrenaline | US & NS-guided | Parasagittal  -1 injection point | 12.7 ± 7.5 minutes,  (motor block onset time: 15.4 ± 7.2 minutes) | 18/20 (90%) at 30 minutes | 571 ± 37.6 minutes |
| A randomised comparative evaluation of supraclavicular and infraclavicular approaches to brachial plexus block for upper limb surgeries using both ultrasound and nerve stimulator.^31^ | Abhinaya et al. | 2017 | RCT | 30mL of 0.5% ropivacaine | US & NS-guided | Parasagittal  -1 injection point | 6.43 ± 4.61 minutes  (motor block onset time: 7.32 ± 2.9 minutes) | 28/30 (93%) at 30 minutes | Not reported |

Supplement 1. Data related to infraclavicular brachial plexus block from previous lieteratures
